# Niosomal Delivery of Celecoxib and Metformin for Targeted Breast Cancer Treatment

**DOI:** 10.3390/cancers15205004

**Published:** 2023-10-16

**Authors:** Haneen A. Basheer, Maram A. Alhusban, Ahlam Zaid Alkilani, Anas Alshishani, Lina Elsalem, Kamyar Afarinkia

**Affiliations:** 1 Department of Pharmacy, Faculty of Pharmacy, Zarqa University, Zarqa 13110, Jordan; 2 Department of Pharmacology, Faculty of Medicine, Jordan University of Science and Technology, Irbid 22110, Jordan; lmelsalem@just.edu.jo; 3 School of Biomedical Sciences, University of West London, London W5 5RF, UK; kamyar.afarinkia@uwl.ac.uk

**Keywords:** breast cancer, metformin, celecoxib, niosomes, nanoparticles

## Abstract

**Simple Summary:**

This study investigates the therapeutic efficacy of combining Metformin (MET) and Celecoxib (CXB) in the treatment of breast cancer. Niosomes, which are drug carriers, were prepared using the thin-film hydration technique. These niosomes were characterized and found to have stable properties when stored at 4 °C for three months. The encapsulated drugs in the niosomes showed increased cytotoxicity compared to their free drug counterparts in both two-dimensional and three-dimensional viability assays. The combination of Metformin Niosomal Particles (MET NPs) and Celecoxib Niosomal Particles (CXB NPs) also led to decreased cell viability in both models. However, the efficacy of the niosomes’ combination was not superior to that of the free drug combination in preventing cell migration. Overall, this study provides valuable insights into the potential application of combining MET and CXB nanoparticle delivery systems for breast cancer treatment.

**Abstract:**

Breast cancer continues to be a prominent worldwide health concern and requires continued investigation into innovative therapeutic approaches. Here, we report the first investigation into the therapeutic efficacy of combining Metformin (MET) and Celecoxib (CXB), both in free and niosomal form, for the treatment of breast cancer. Our investigation encompassed the characterization of these niosomal drug carriers, their stability assessment, and their effect on breast cancer cell models. The thin-film hydration technique was employed to prepare niosomes with spherical, uniform-size distributions and high encapsulation efficiencies. The niosomes were characterized by TEM, particle size analyzer, and ATR-FTIR. The niosomes with an average size of 110.6 ± 0.6 and 96.7 ± 0.7, respectively, for MET and CXB were stable when stored at 4 °C for three months with minimal drug leakage, minor changes in encapsulation efficiency and size, and unchanged physicochemical parameters. Evaluation in two-dimensional (2D) and three-dimensional (3D) viability assays demonstrated an increased cytotoxicity of encapsulated drugs when compared to their free-drug counterparts. Additionally, the combination of Metformin Niosomal Particles (MET NPs) and Celecoxib Niosomal Particles (CXB NPs) led to decreased cell viability in both 2D and 3D models compared to each drug administered individually. When comparing the effect of the niosomal versus the free combination of the drugs on cell migration, we found that both interventions effectively prevented cell migration. However, the efficacy of the niosomes’ combination was not superior to that of the free drug combination (*p* < 0.05). In conclusion, the results of this study provide valuable insights into the potential application of combining MET and CXB nanoparticle delivery systems to breast cancer treatment. Exploring the in vivo application of this drug delivery system could open new avenues for more effective and targeted therapeutic approaches for breast cancer patients.

## 1. Introduction

Breast cancer is a highly prevalent and potentially lethal disease that significantly impacts women’s health globally [1]. The management of breast cancer often employs combining multiple approaches, including surgery, radiation therapy, cytotoxic and targeted chemotherapy, and hormone therapy [2]. The selection of treatment modalities depends on various factors, such as the stage and type of breast cancer, the presence of hormone receptors, and the patient’s general health.

Despite the advances in cancer research and the wide range of therapeutic options currently available, there are still persistent hurdles in the development of effective and selective drugs for breast cancer. In addition, the use of existing chemotherapeutic agents for breast cancer is limited by many factors such as loss of efficacy due to the development of drug resistance, inadequate selectivity, and the occurrence of adverse effects [3]. For this reason, there is an urgent need for novel therapeutic approaches, either alone or in combination. Indeed, the investigation of targeted combination therapies has gained considerable attention as a potential avenue to further optimize breast cancer treatment in recent years [4].

The repurposing of drugs in breast cancer has emerged as a promising strategy to increase available therapeutic options and enhance patient survival. This approach accelerates the drug development process, leveraging established agents’ existing safety profiles and providing novel and cost-effective treatment options for breast cancer patients. For instance, CXB, a nonsteroidal anti-inflammatory drug, and MET, a widely used medication for type 2 diabetes, have been investigated as potential candidates for repurposing due to their established pharmacological properties and safety profiles. Preclinical studies have demonstrated that CXB can suppress breast cancer growth through its anti-inflammatory effects [5]. Similarly, MET has shown promising anti-cancer properties through its impact on cell signaling pathways and metabolism [6]. Furthermore, nanoparticles have emerged as a promising tool in breast cancer treatment, attracting significant attention due to their potential to enhance therapeutic outcomes while minimizing side effects. The nanoparticle drug delivery systems enable the targeted and selective delivery of therapeutic agents to breast cancer cells, thereby improving treatment efficacy [3]. By encapsulating drugs within nanoparticles, the formulations can overcome biological barriers, improve pharmacokinetic characteristics, and enhance drug penetration in the tumor microenvironment, and address many of the challenges associated with conventional cancer therapies [7]. In recent years, there has been a growing interest among researchers in the field of drug delivery systems regarding the utilization of niosomes as a means of targeted drug delivery [8]. Niosomes, resembling liposomes in structure, are constructed using non-ionic surfactants and have the ability to transport drugs that are soluble in water as well as drugs that have low solubility in water [9,10,11]. Nevertheless, niosomes exhibit greater stability in both formulation and storage when compared to liposomes [12]. In addition, the ability to tailor their surface or modify their composition makes it feasible to attain the desired pharmacokinetic attributes, enabling controlled drug release and consequently resulting in reduced toxicity, enhanced targeting, and improved drug bioavailability [13]. Moreover, the manufacturing of niosomes is simple and can be scaled up cost-effectively [14]. In this study, we investigate for the first time the potential of a nanoparticle-based therapy by combining two well-established drugs, CXB and MET, as a novel therapeutic approach for breast cancer. Both drugs have individually demonstrated promising anti-cancer effects in preclinical studies, and their safety profiles are well-characterized in clinical settings [15,16]. This work represents both the initial evaluation of combining MET and CXB in the context of breast cancer and the first instance of utilizing their nanoparticles’ formulation combined for cancer treatment. By combining their mechanisms of action, we aim to uncover a new treatment modality that will improve the treatment options for cancer patients.

Furthermore, the utilization of the 3D spheroid model is increasingly recognized as a crucial instrument in the field of cancer research due to its ability to represent an intermediate level of complexity, bridging the gap between 2D monolayer models and in vivo solid tumors [17]. Furthermore, these 3D spheroids offer a better tool for assessing the efficacy of nanoparticles compared to 2D experimentation. Consequently, our objective is to evaluate the efficacy of our combinational therapeutic approach in a 3D model in order to obtain insights into the in vivo behavior of these medications.

## 2. Materials and Methods

### 2.1. Materials

MET HCL and CXB were generously donated by Dar al Dawa Pharma (Amman, Jordan). Chloroform, diethylamine, triethylamine, as well as HPLC-grade acetonitrile, methanol, and water were purchased from Tedia (Fairfield, OH, USA). A phosphate buffered saline (PBS) tablet, Span^TM^ 60, and cholesterol were purchased from Sigma Aldrich (Dorset, UK). Ammonium acetate ≥97.0% ACS was purchased from VWR Chemicals BDH^®^, glacial acetic acid from Honeywell^®^ (Raunheim, Germany), Penicillin-Streptomycin Solution 100× from Euroclone^®^ (Milan, Italy), RPMI 1640 medium with L-Glutamine from Caisson Labs (Smithfield, UT, USA), Dulbecco’s Phosphate Buffered Saline from Euroclone^®^ (Milan, Italy), L-Glutamine 100X 200 mM from Euroclone^®^ (Milan, Italy), Sodium Pyruvate Solution from Eurobio^®^ Scientific (Les Ulis, France), Cytiva^®^ HyClone Fetal Bovine Serum from Global Life Sciences^®^ (Pasching, Austria), CellTiter-Glo^®^ 3D Cell Viability Assay from Promega^®^ (Mannheim, Germany), Thizolyl Blue Tetrazolium Bromide Powder BioReagent from Sigma-Aldrich Inc.^®^ (St. Louis, MO, USA), dimethyl sulfoxide (DMSO) from Sigma-Aldrich Inc.^®^ (St. Louis, MO, USA), and ROS-Glo™ H_2_O_2_ assay from Promega^®^ (Mannheim, Germany).

### 2.2. Methods

#### 2.2.1. Preparation of Niosomes

**Figure 1:**
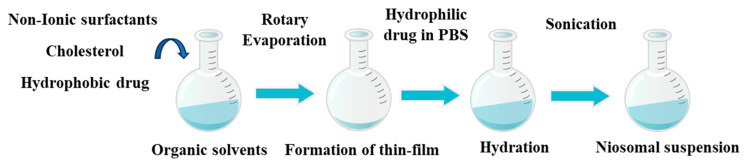
Schematic illustration of the formulation of MET and CXB niosomes using the thin-film hydration method.

The preparation of niosomes was performed using the thin film hydration method. Niosomes loaded with MET and CXB were formulated by dissolving Span 60 and cholesterol or Span 60, cholesterol, and Tween 80, as presented in Table 1, in a 10 mL organic solvent (chloroform: methanol) (80:20) in a 100 mL round-bottomed flask. The organic solvent was then evaporated under reduced pressure using a rotary evaporator to form a thin film. The film was hydrated with 15 mL PBS (pH = 7.4) containing MET to prepare MET NP. For CXB NP, the drug was dissolved in the organic solvent used to form the thin film due to its hydrophobic properties. Then, hydration of the film was performed using only PBS (pH = 7.4), as shown in Figure 1. The niosomes were sonicated for 15 min and then stored at 4 °C in a refrigerator for 24 h before further characterization.

#### 2.2.2. Characterization of Niosomes Using Transmission Electron Microscope (TEM)

The morphology of the niosomes was examined using TEM, which was equipped with a Mega View II^®^ digital camera. The niosomes were diluted in distilled water at a volume-to-volume ratio of 1:2% and afterward applied onto a carbon-coated copper grid. The samples were then let to dry before being subjected to imaging. ImageJ^®^ 154a software was employed to analyze the niosomes’ morphology.

#### 2.2.3. Measurement of Particle Size (PS), Zeta Potential (ZP), and Polydispersity Index (PDI)

The particle size (PS) and polydispersity index (PDI) of the chosen formulation were assessed utilizing dynamic light scattering (DLS) through a Brookhaven 90-plus particle size analyzer (Holtsville, NY, USA). The electrophoretic light scattering (ELS) technique was employed for the measurement of the zeta potential (ZP) of particles. The sample was prepared by dispersing 50 µL of niosomal suspension in 950 µL of distilled water at 25 °C.

#### 2.2.4. Purification of Niosomes

The optimization of the purification method for niosomal drugs was achieved by investigating the kinetics of drug movement through a dialysis membrane with a molecular weight cutoff of 12–14 kDa. For MET NP purification, a drug solution was prepared by dissolving 100 mg of MET in 15 mL PBS, and the membranes were filled with 1 mL of the solution and immersed in beakers with 50 mL PBS while being stirred. HPLC-UV analysis of the samples taken at various time intervals over four hours determined the time required to remove all free drugs from the dialysis bag. The same method was applied to evaluate the purification of CXB NP, where 10 mg of CXB NP was dissolved in 15 mL of methanol, and 1 mL of the solution was transferred to the dialysis bag immersed in 200 mL of PBS. Samples were taken and analyzed to determine the optimal time for the purification step.

#### 2.2.5. Drug Entrapment Efficiency

After the purification process, the MET in its free form was removed from the dialysis membrane, leaving pure MET NP inside the dialysis bag. To determine the drug encapsulation efficiency (EE) of MET NPs, a 1 mL sample was collected from the dialysis medium at the 1 h mark and diluted with PBS to a final volume of 10 mL. Subsequently, the sample was subjected to HPLC with ultraviolet detection (HPLC-UV) to quantify the EE, as follows:

$$\%EE=\left(Total\;amount\;of\;MET-Free\;amount\;of\;MET\right)/\left(Total\;amount\;of\;MET\right)\times 100\%$$

Here, “Total amount of MET” denotes the initial quantity of MET used for the preparation of MET NPs, while “Free amount of MET” represents the portion of MET that remained unencapsulated within the niosomal formulation.

For the purification of CXB, the process required 2 h, after which the contents of the dialysis bag were transferred to a centrifuge tube. A 100 μL sample of purified niosomes was solubilized with 10 mL of isopropyl alcohol, sonicated for 15 min, and subsequently analyzed using HPLC-UV. A 1 mL sample was withdrawn from the solution for analysis. The EE of CXB was calculated using the following equation:

$$\%EE=\left(Entrapped\;amount\;of\;CXB\right)/\left(Total\;amount\;of\;CXB\right)\times 100\%$$

In this equation, “Entrapped amount of CXB” represents the quantity of the drug effectively encapsulated within the niosomes, while “Total amount of CXB” signifies the total amount of CXB utilized during the preparation process.

#### 2.2.6. Attenuated Total Reflectance—Fourier-Transform Infrared Spectroscopy (ATR-FTIR)

The formulated MET NP (M2) and CXB NP (O2) were separated from the unentrapped drug and subjected to freeze drying at −50 °C for 48 h after overnight freezing at −80 °C. The resulting white powder niosomes were stored in a tightly sealed glass container at 4–8 °C.

The Perkin Elmer UATR-II instrument (Waltham, MA, USA) was utilized to conduct ATR-FTIR spectroscopy in order to evaluate potential incompatibilities. The samples underwent examination in the wavenumber range of 4000–400 cm^−1^ with a resolution of 2 cm^−1^, capturing 32 scans for each sample. The spectrum data obtained were subjected to analysis using Ira^®^ FTIR data explorer V 1.0 software. The ATR-FTIR technique was utilized to analyze the spectra of MET, CXB, and Span 60, Cholesterol and the physical mixture of the components were used for niosomal formulations and the blank niosomal formulations.

#### 2.2.7. In Vitro Drug Release Study

In vitro release of the (M2) MET NP and (O2) CXB NP was assessed using a dialysis membrane with a molecular weight cutoff (MWCO) of 12–14 kDa as described [8]. Prior to the release study, the dialysis membrane was pre-soaked overnight in PBS with a pH of 5.1. Each niosomal formulation (1 mL) was then placed inside a dialysis bag and submerged in a beaker containing 100 mL of PBS at the same pH. The entire setup was incubated in a shaker incubator at 37 °C for 72 h. At specific time intervals (1, 2, 3, 4, 5, 24, 30, 48, 54, and 72 h), 5 mL samples were collected from the outer release medium, and an equal volume of fresh PBS (pH = 5.1) was added to maintain sink conditions. These collected samples were subjected to analysis using HPLC-UV to determine the release profiles of MET NP and CXB NP over the specified time points.

#### 2.2.8. Stability Study

A short-term stability assessment was performed to evaluate the alterations in PS, PDI, and EE% of the optimal niosomal formulations (M2 and O2) over a duration of four months. These formulations were selected based on their characterization results. Throughout the study, the desired formulations were stored at a temperature of 4 °C.

#### 2.2.9. Chromatographic Method of Analysis of MET NP and CXB NP

A Shimadzu HPLC system (Tokyo, Japan) equipped with a SIL-20A autosampler, SPD-20A detector, CTO-20A temperature regulator oven, and LC-20AT pump was used for the quantification of both MET NP and CXB NP. Chromatographic separation of MET NP was achieved using a Zorbax C8 column (250 × 4.6 mm, 5 μm) from Agilant^®^ (Santa Clara, CA, USA), and the mobile phase was set to flow at a rate of 1.0 mL/min. The injection volume for HPLC analysis was 10 μL, and the drug detection was conducted at λ_max_ 233 nm. The HPLC analysis was performed at 25 °C, and the mobile phase consisted of a mixture of Acetoni-trile and 0.02 M Ammonium Acetate Buffer at a ratio of 80:20, *v*/*v*%. In contrast, the chromatographic separation of CXB NP was performed utilizing the aforementioned column, with the mobile phase being adjusted to a flow rate of 1.5 mL/min. The injection volume utilized for HPLC analysis was 20 μL, while the detection of the drug was performed at a wavelength of maximum absorption (λ_max_) of 253 nm. The temperature was adjusted to 25 °C, and the mobile phase consisted of a mixture of the Acetoni-trile and the 0.02 M Ammonium Acetate Buffer at a ratio of (80:20, *v*/*v*%).

#### 2.2.10. Cell Culture

The MCF-7 and MDA-MB-231 cell lines used in this study were obtained from the American Type Culture Collection (ATCC), Manassas, VA, USA.

The MCF-7 and MDA-MB-231 cell lines were cultivated and maintained in a humidified atmosphere at 37 °C with 5% CO_2_. The medium used in this study was RPMI 1640, supplemented with 10% *v*/*v* fetal bovine serum (FBS), 2 mM L-glutamine, 1 mM sodium pyruvate, and 1% *v*/*v* Penicillin (10,000 U/mL)—Streptomycin (10 mg/mL).

#### 2.2.11. MTT Cell Viability Assay

MCF-7 and MDA-MB-231 breast cancer cells were seeded in a 96-well plate at a concentration of 5 × 10^3^ cells/mL and were allowed to attach and grow for 24 h. Subsequently, the cells were treated for 72 h with varying concentrations of MET, MET NP (50–2.5 mM), CXB, and CXB NP (100–5 μM) to compare the free and nanoparticle drug forms. Cell viability was assessed using the MTT assay (Sigma, St. Louis, MO, USA) following the manufacturer’s instructions. Briefly, after the treatment period, 20 μL of the MTT solution (5 mg/mL) was added to each well, and the plate was incubated at 37 °C for 4 h. Following this, the aqueous solution was removed, cells were lysed using DMSO, and the optical density of each well was measured at 570 nm. The percentage of viable cells was calculated relative to the untreated control cells, enabling the assessment of the inhibitory effects of different drug concentrations on cell viability. To construct dose-response curves for each drug in both its free form and niosomal formulation, a comprehensive range of concentrations was tested to evaluate the inhibitory effects on proliferation. Non-linear regression analysis with curve fitting using GraphPad Prism Version 9.0.0 was employed to determine the IC_10_, IC_20_, and IC_50_ values for each compound representing the drug concentrations causing 10%, 20%, and 50% inhibition of the biological response, respectively.

#### 2.2.12. Spheroid Formation and Viability Assay

A suspension of MCF-7 cells, consisting of 20% methylcellulose (MC), was introduced into the wells of an ultra-low attachment plate at concentrations of 2000 and 10,000 cells per well. The plate underwent centrifugation at a speed of 1000 (rcf) for a duration of 10 min. After a period of 24 h, spheroids were formed and monitored for growth by measuring the diameter for 7 days.

In order to assess the viability of spheroids inside the 3D cultures, we utilized the Cell Titer-Glo^®^ 3D Cell viability assay (Promega, G9681), a specialized assay built expressly for this objective.

Following a 72 h period of spheroid formation, we administered different treatments, namely MET (ranging from 2.5 to 100 millimolar) and MET NP (ranging from 2.5 to 50 millimolar), as well as CXB (ranging from 10 to 200 micromolar) and CXB NP (ranging from 10 to 100 micromolar). Control groups, including untreated spheroids and spheroids treated with blank niosomes at equivalent drug concentrations, were also used. The plate was incubated for 72 h to facilitate the attainment of the utmost drug effect.

Following the incubation period, the spheroids were transferred to white opaque-walled 96-well plates, and the CellTiter-Glo^®^ reagent was added. Subsequently, the luminescence signal was measured using the Glomax^®^ Multi detection system. Viability was calculated using the formula:

(A−B)/(C−B)×100%

where A represents the luminescence of treated spheroids, B is the luminescence of blank media, and C is the luminescence of untreated spheroids.

The IC_10_, IC_20_, and IC_50_ values for each compound were established by non-linear regression analysis with curve fitting, and GraphPad Prism Version 9.0.0 was utilized to ascertain these values for each compound.

#### 2.2.13. Spheroids Penetration Assay

In order to verify the penetration of drugs into the spheroids, drugs were introduced into the wells of 3-day-old spheroids at concentrations of 100 mM MET, 100 mM MET NP, 100 µM CXB, or 100 µM CXB NP. To accurately measure the quantity of drug that did not enter the spheroids, we collected 100 µL samples from the surrounding media at specific time intervals: 1, 2, 3, 4, 24, 48, and 72 h.

In order to eliminate any potential influence from the media components, the collected samples were diluted with acetonitrile and subsequently underwent a 15 min centrifugation at a speed of 2000 rpm. Afterward, the supernatant was collected and mixed with isopropyl alcohol to break the niosomes apart and retrieve the remaining drug trapped inside them. This allowed us to obtain a suitable concentration for later analysis using HPLC. HPLC analysis quantified the non-penetrated drug amount, allowing for the calculation of drug penetration by subtracting this quantity from the initially added 100% of the drugs.

#### 2.2.14. Wound Healing Assay

The anti-migratory properties of various formulations were assessed using the wound healing assay. Briefly, the breast cancer cell line MCF-7 was cultured in a 24-well plate at a seeding density of 8 × 10^5^ cells/mL in 2% FBS media. After 24 h, a uniform scratch wound was created using a 200 µL pipette tip, and the cells were washed to remove any debris.

The treatment groups consisted of MCF-7 cells exposed to IC_10_ concentrations of MET (0.3 mM) and CXB (1.0 µM) individually, as well as a combination of MET and CXB at concentrations of (0.3 mM and 1.0 µM), respectively. Additionally, MET NP (0.2 mM), CXB NP (0.7 µM), and a combination of MET NP and CXB NP 0.2 mM and 0.7 µM, respectively, were examined. Hydrogen peroxide (H_2_O_2_) was introduced at a concentration of 200 µM to enhance cell migration and mimic wound healing conditions [18]. Control wells included 2% FBS media alone as a negative control and H_2_O_2_ (200 µM) alone as a positive control. Images of the wound area were captured at 0 and 48 h using an OPTIKA^®^ Inverted Microscope IM-3 (Ponteranica, BG, Italy). ImageJ 154a software was used to quantify and measure the wound’s free surface area at two different time points. To assess cell migration, we determined the percentage of free surface area after migration using the following formula:

$$\%\;of\;free\;surface\;area\;after\;migration=100-\left(\frac{A-B}{A}\right)\times 100$$

where A corresponds to the free surface area at 0 h and B denotes the free surface area after migration to the particular time point.

#### 2.2.15. Statistical Analysis

The statistical analysis was conducted using either GraphPad Prism software version 9.0.0 or Microsoft Excel. The studies were performed in triplicate, and the findings are reported as means ± standard deviation (SD). The means were compared using a two-tailed Student’s *t*-test, and *p*-values were computed to evaluate the statistical significance of the findings. In this study, statistical significance was represented by an asterisk (*) when the *p*-value was less than 0.05 and by two asterisks (**) when the *p*-value was less than 0.01.

## 3. Results

### 3.1. Characterization of MET- and CXB-Loaded Niosomes

**Figure 2:**
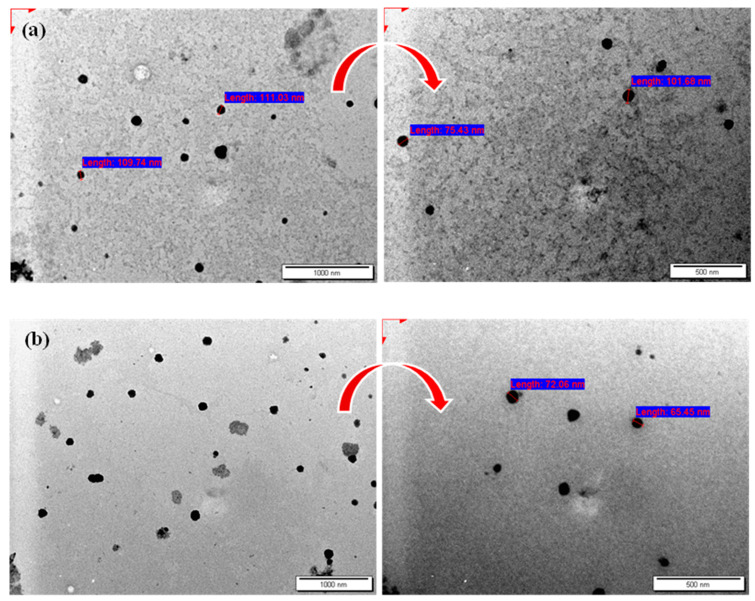
TEM micrographs of (**a**) MET NP (M2) and (**b**) CXB NP (O2). Arrows represent enlargement of the image at 50 Kx.

The thin film hydration approach was successfully employed to prepare several formulations of either niosomal formulations loaded with MET or CXB. The morphology of the developed niosomes was validated using TEM, as depicted in Figure 2. The niosomes displayed a spherical morphology, providing evidence of their effective development. Furthermore, the particle size analyzer findings presented in Table 2 revealed that the particle size of both M2 and O2 niosomal formulations was similar to those obtained using TEM. Previous studies have noted that the particle size measured by TEM may exhibit similarities to the dynamic light scattering approach of a particle size analyzer [11,19].

Regarding particle size (PS), the MET NP formulations displayed a size range from 110.6 ± 0.6 nm to 129.5 ± 3.1 nm. Notably, M2 exhibited the smallest particle size, with statistical significance observed compared to both M1 (*p* < 0.01) and M3 (*p* < 0.01). In contrast, the CXB NP formulations demonstrated PS values ranging from 96.7 ± 0.7 nm to 159.1 ± 1.7 nm. Interestingly, O2 displayed the smallest particle size, with statistical significance compared to O1 (*p* < 0.01) and O3 (*p* < 0.01).

The ZP values observed for MET NP varied between −42.15 ± 3.00 and −56.18 ± 1.89, but for CXB NP, the range was between −50.43 ± 0.785 and −53.93 ± 1.55. The ZP values observed in this study are deemed sufficient for inducing strong repulsive interactions between the niosomes, preventing their aggregation and preserving the stability of the vesicles.

### 3.2. Purification of Niosomes

The dialysis method is employed as a purification technique to separate the formulated niosomes from the unencapsulated drug. This method relies on the principles of diffusion and osmosis and is facilitated by the use of a semi-permeable membrane [20].

**Figure 3:**
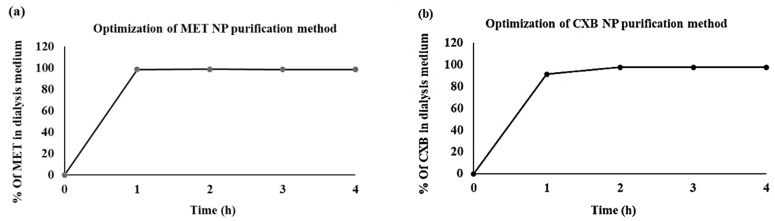
Optimization of the niosome purification methods for (**a**) MET NP and (**b**) CXB NP.

We conducted an optimization process to ascertain the duration required for the complete removal of the free drug quantity from the dialysis medium. The findings indicated that the duration required for the absence of any wash-off of MET was 1 h, whereas it took 2 h for CXB, as illustrated in Figure 3. This indicates the purity of the formulated niosomes.

### 3.3. Drug Entrapment Efficiency

Table 3 displays the percentage encapsulation efficiency (EE) of the niosomal formulations. Among the various formulations of MET, it was observed that formula M2 demonstrated the best encapsulation efficiency (EE) with a value of 68.94 ± 1.28%. On the other hand, for CXB, the formulation O2 exhibited the highest EE of 94.44 ± 2.09%.

### 3.4. Attenuated Total Reflectance—Fourier-Transform Infrared Spectroscopy (ATR-FTIR)

**Figure 4:**
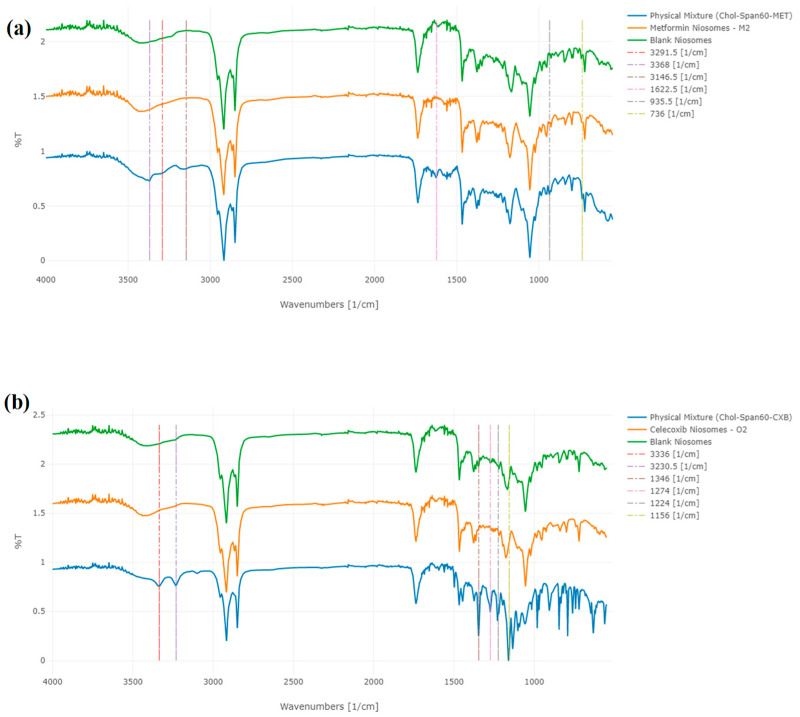
ATR-FTIR analysis of (**a**) MET NP (M2), blank NP, and the physical mixture of M2 components and (**b**) CXB NP (O2), blank NP, and the physical mixture of O2 components. Labels present the characteristic peaks of MET and CXB.

The assessment of compatibility between MET or CXB and the additional components used during the development of the optimal niosomal formulations, M2 and O2, respectively, was conducted by ATR–FTIR analysis, as depicted in Figure 4. The IR spectra display the primary absorption bands for cholesterol, Span 60, MET, and CXB, which are summarized in Table 4.

The infrared spectra exhibited the primary absorption bands for MET, which included two typical bands at 3291.5 cm^−1^ and 3368 cm^−1^, corresponding to the N–H primary stretching vibration. Additionally, a band at 3146.5 cm^−1^ was observed and attributed to the N–H secondary stretching. Another noteworthy feature was the presence of a characteristic band at 1622.5 cm^−1^, which was assigned to C-N stretching, as reported earlier by Jagdale et al. (2011) and Samed et al. (2018) [21,22]. Furthermore, two bands at 935.5 cm^−1^ and 736 cm^−1^ were identified, as documented by Kenechukwu et al. [23].

In the case of CXB, the primary peaks were observed at 3336 cm^−1^ and 3230.5 cm^−1^, indicating the stretching of N-H2 bonds. Additionally, a band at 1346 cm^−1^ was observed, corresponding to the stretching of S=O bonds. Two further bands were identified at 1274.5 cm^−1^ and 1229 cm^−1^, which can be attributed to the stretching vibrations of C–F bonds. Lastly, another band associated with the stretching of S=O bonds was detected at 1156 cm^−1^, as previously reported by Lakshmi et al. and Vijayakumar et al. [24,25].

The primary spectral peaks of Span 60 were detected at around 3400 cm^−1^ as a result of OH stretching, 2917 cm^−1^ suggesting -CH stretching, and a notable peak at 1736 cm^−1^, signifying the robust C=O ester bond. Farmoudeh et al. and Ur Rehman et al. have reported the presence of supplementary peaks at 1174 cm^−1^, which can be attributed to the stretching vibrations of C–O and C–C bonds [26,27]. Furthermore, another peak was identified at 721 cm^−1^, which belongs to C–C connections. In contrast, cholesterol exhibits distinct spectral peaks at specific wave numbers. Notably, a peak is observed at 3430.5 cm^−1^, which corresponds to the stretching of O-H bonds. Additionally, peaks at 2931 cm^−1^ and 2867 cm^−1^ are attributed to the stretching of C-H bonds. Furthermore, vibrations associated with C-O bending are evident at 1055 cm^−1^. Finally, two peaks at 958.5 cm^−1^ and 840.5 cm^−1^ are observed, which can be attributed to aromatic substitutions, as presented by Samed et al. and Zaid Alkilani et al. [9,22].

In Figure 4, a prominent characteristic band appears in the region of 3400 cm^−1^ in MET, CXB, and blank niosomes. This signal arises from the OH stretching vibration of cholesterol and Span 60, indicating their interaction and bonding in the formation of the niosomal structure. Interestingly, none of the characteristic peaks for CXB are observed in its corresponding niosomal formulations. Conversely, in MET niosomes, only minor shifts from 736 cm^−1^ to 742 cm^−1^ are evident, signifying an interaction between MET and the formulated niosomes through bond formation. This absence of most of the peaks in the case of MET can be attributed to the entrapment of the drugs within the core of the niosomes. No free drug molecules are detected on the surface of the niosomes due to the optimized washing process designed to completely eliminate unentrapped molecules. Additionally, the weight of the drug loaded, whether MET or CXB, is minimal compared to the total formula weight (MET accounts for approximately 12.5% and CXB accounts for approximately about 1.25% of the total formula), which explains the absence of all CXB peaks.

To further confirm the niosomal formulation in M2 niosomes, characteristic cholesterol peaks shifted from 2931 cm^−1^ to 2937 cm^−1^, from 2867.5 cm^−1^ to 2870.5 cm^−1^, and from 481.5 cm^−1^ to 844.5 cm^−1^. Similarly, Span 60 characteristic peaks shifted from 3400 cm^−1^ to 3391 cm^−1^, from 1736 cm^−1^ to 1739.5 cm^−1^, and from 1174.5 cm^−1^ to 1177.5 cm^−1^.

For the confirmation of niosomal formulation in O2 niosomes, characteristic cholesterol peaks shifted from 2867.5 cm^−1^ to 2871.5 cm^−1^ and from 840.5 cm^−1^ to 844.5 cm^−1^, while Span 60 characteristic peaks shifted from 3400 cm^−1^ to 3391.5 cm^−1^ and from 1735.5 cm^−1^ to 1738.5 cm^−1^.

### 3.5. In Vitro Drug Release Study

This study aimed to evaluate the release profiles of MET NP (M2) and CXB NP (O2) under two distinct pH conditions: an acidic pH of 5.1 and a physiological pH of 7.4. These pH values were deliberately chosen to enable a comparative analysis of drug release behavior. This choice stemmed from previous research indicating that cancer cells typically exhibit a lower pH environment compared to the typical pH of 7.4 found in healthy cells [28]. It is important to note that niosomes, the vesicular structures used in this study, have a propensity to undergo swelling or breakdown more readily under acidic conditions. Additionally, surfactants experience a higher rate of hydrolysis in acidic environments compared to the typical pH found in the body [29,30].

**Figure 5:**
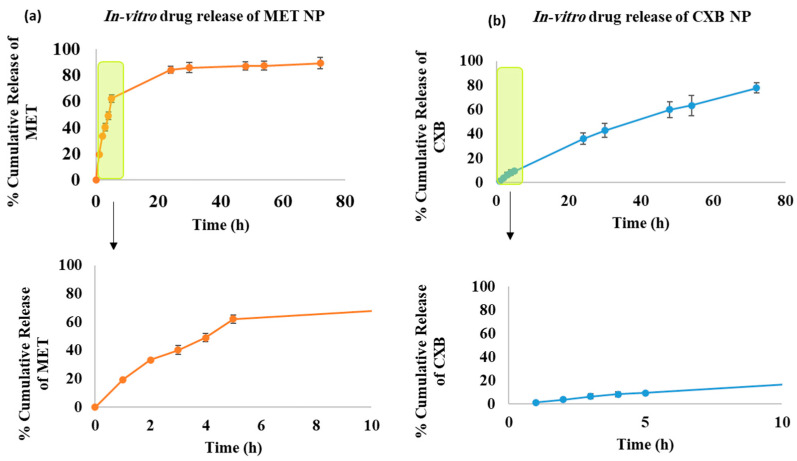
In vitro drug release of (**a**) MET NP (M2) at pH 5.1 and (**b**) CXB NP (O2) at pH 5.1. The Red and blue colors represent the % of cumulative release of MET and CXB, respectively. The green box demonstrates the first 8 h of the release interval.

These specific formulations, namely M2 and O2, were chosen due to their higher percentage of EE, indicating their greater ability to retain the drugs. The release characteristics of both medications over a 72 h span at pH 5.1 are shown in Figure 5. It is worth noting that although the niosomal vesicles were made using the same compositions, there was a noticeable difference in the way the two formulations released their contents. During the first 5 h of the release study, 62.12% of MET was released from the M2 formulation, whereas only 9.43% of CXB was released from the O2 formulation. The difference in release can mainly be attributed to the individual characteristics of the drugs involved, such as their polarity and molecular weight.

It has been previously shown that niosomes containing MET release the drug at a slower rate compared to the free drug solution [31]. The release of MET from niosomes can be described as having two distinct phases. Initially, there is a rapid release that lasts for about 1–5 h. This is followed by a slower but continuous release, which continues for the entire 72 h and can reach up to 89.2% of the total amount of MET. However, the release of CXB from NP was slower in the first 5 h compared to MET from MET NP release, but over the entire 72 h, it resulted in a total release of 77.80% by the end of the interval. Furthermore, when comparing the release at pH 5.1 to pH 7.4, it became evident that the release profiles at pH 7.4 were generally slower and did not surpass the release observed at pH 5.1 at any of the measured time points. At the end of the 72 h evaluation period, MET NP reached a maximum release level of 66.81%, while CXB NP exhibited a maximum release of 33.90% duration (Supplementary Figure S1).

### 3.6. Stability Study

The behavior of nanocarriers in vitro and in vivo is influenced by their stability [8]. Hence, an assessment was conducted to evaluate the short-term stability of the niosomal formulations with the most optimal drug entrapment efficiencies (M2 and O2) over a duration of three months. The evaluation was centered on the observation of alterations in EE, PS, and PDI of the niosomes that were stored at a temperature of 4 °C.

During a three-month storage period, it was observed that there was an acceptable amount of drug leakage in M2. The entrapment efficiency (EE) of M2 had a modest reduction from 68.94 ± 1.28 to 57.12 ± 0.95 (*p* < 0.05). In contrast, the O2 niosomal formulation exhibited negligible leakage, as evidenced by the lack of a significant disparity in EE from 94.54 ± 2.09 to 90.73 ± 3.26 (*p* > 0.05) between the freshly prepared formulation and the one stored for three months (Table 5). Regarding PS, O2 niosomes exhibited negligible alterations, but M2 niosomes demonstrated a marginal increase in their average size. The PDI values for both formulations were found to be less than 0.3, indicating that the vesicles were uniformly distributed and the niosomes still exhibited stability. Furthermore, no substantial changes were noted in the physicochemical parameters, such as visual characteristics, and no formation of precipitation was apparent throughout the duration of storage. The results presented in this study offer evidence of the remarkable physical stability of both M2 and O2 formulations when stored at a temperature of 4 °C for the entire length of three months.

### 3.7. Effect of the Combination of MET NP and CXB NP on Cell Viability in a Monolayered Cell Culture

We investigated the viability of monolayered MCF-7 and MDA-MB-231 breast cancer cells treated with free MET and MET NP, as well as free CXB and CXB NP, at various concentrations. Viability was assessed at 10 μM, 50 μM, and 100 μM for free CXB and CXB NP, and at 2.5 mM, 5 mM, and 10 mM for free MET and MET NP.

**Figure 6:**
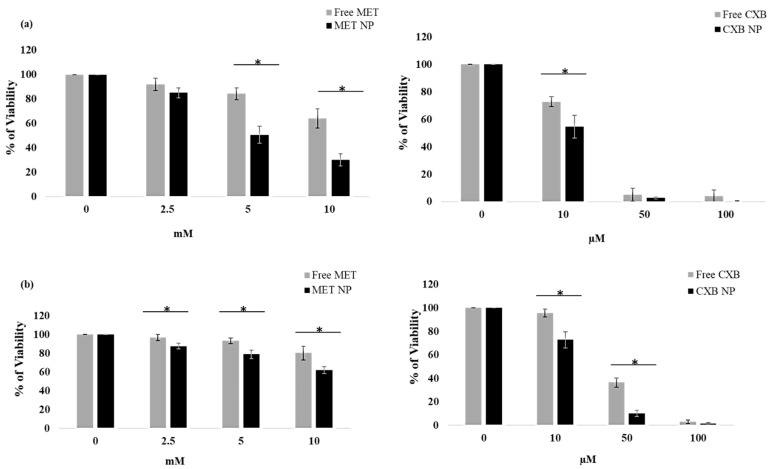
Effect of MET free, MET NP, CXB free, and CXB NP on MCF-7 (**a**) and MDA-MB-231 (**b**) cell lines compared to the control, where no drug is added. Values are the mean of three independent experiments, and error bars represent the standard deviation (SD). * *p* < 0.05.

Both free MET and MET NP showed a concentration-dependent decrease in MCF-7 cell viability (Figure 6a). However, at all tested concentrations, MET NP exhibited significantly lower cell viability compared to its free counterpart. Notably, at the highest concentration (10 mM), free MET resulted in a viability of 64.02%, while MET NP showed a relatively lower viability of 30.2% (*p* < 0.05). Similarly, for MDA-MB-231 cells, free MET and MET NP displayed concentration-dependent effects on cell viability (Figure 6b). Consistent with the results for MCF-7 cells, MET NP outperformed free MET, showing significantly lower cell viability at all concentrations. At 10 mM, free MET exhibited a viability of 80.37%, whereas MET NP showed 62.44% viability (*p* < 0.05).

In regard to CXB treatments, in MCF-7 cells (Figure 6a), both free CXB and CXB NP exhibited a concentration-dependent reduction in cell viability. CXB NP showed a substantially lower cell viability compared to free CXB. At 100 μM, free CXB resulted in a viability of 4.08%, whereas CXB NP showed an even lower viability of 0.33% (*p* < 0.05).

Similarly, for MDA 231 cells, free CXB and CXB NP displayed concentration-dependent effects on cell viability (Figure 6b). Furthermore, CXB NP showed slightly improved cell viability compared to free CXB at all concentrations. At 100 μM, free CXB exhibited a viability of 3.18%, while CXB NP showed 1.59% viability (*p* < 0.05). These results imply that the effect of nanoparticle delivery on MET and CXB efficacy might vary between different breast cancer cell lines.

Subsequently, we performed an MTT assay to generate dose-response curves for each drug and determine the IC_10_, IC_20_, and IC_50_ values, which are vital for potential combination therapies. The outcomes revealed that the nanoparticle formulations (MET NP and CXB NP) demonstrated higher cytotoxicity at lower concentrations compared to the free drugs (Table 6). Notably, in MCF-7 cells, MET NP exhibited lower IC10 and IC20 values than free MET, signifying improved efficacy at lower, non-lethal doses. Similarly, in MCF-7 cells, CXB NP displayed a comparable trend of enhanced cytotoxicity at sub-lethal doses in comparison to free CXB. Moreover, in MDA-MB-231 cells, both MET NP and CXB NP showed lower IC10 and IC20 values than their respective free drugs, indicating increased cytotoxicity at lower concentrations.

**Figure 7:**
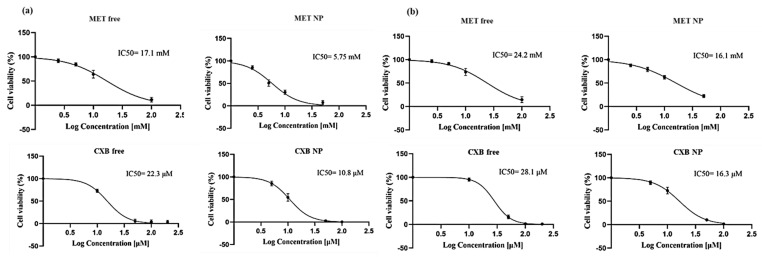
The dose-response curve of MET free, MET NP, CXB free, and CXB NP in MCF-7 (**a**) and MDA-MB-231 (**b**) cell lines. Values are the mean of three independent experiments, and error bars represent the standard deviation (SD).

The results of this study indicate that drug delivery systems utilizing nanoparticles exhibit considerable potential in enhancing the effectiveness of drugs and optimizing the treatment of breast cancer. Figure 7 and Table 6 display the data that provide a summary of the dose-response curves and IC values for each formulation in both cell lines.

**Figure 8:**
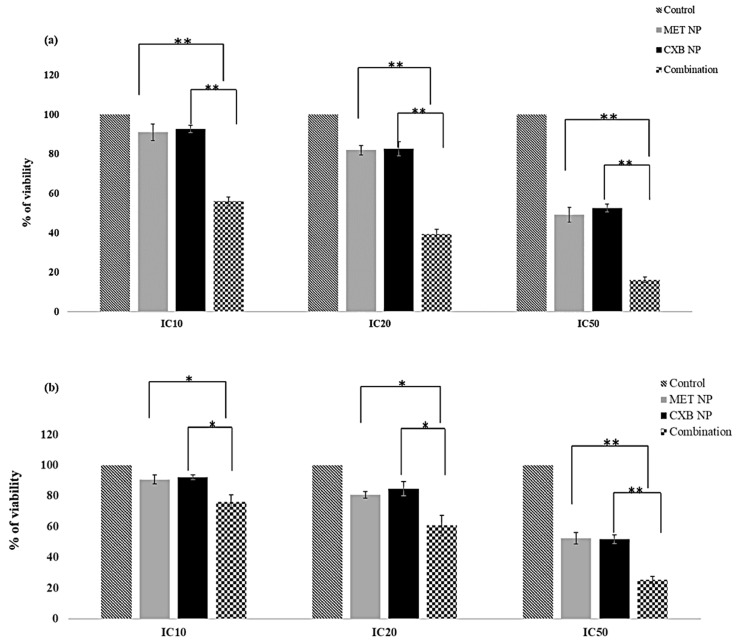
Effect of Met NP and CXB NP in combination between IC_10_, IC_20_, and IC_50_ on the viability of (**a**) MCF-7 and (**b**) MDA-MB-231 cell lines. Values are the mean of three independent experiments, and error bars represent the standard deviation (SD). * *p* < 0.05 and ** *p* < 0.01.

Considering the superior responses of nanoparticle formulations at lower concentrations (Table 6), we sought to explore the potential improved effects of combining MET NP and CXB NP treatments in comparison to each drug alone. The viability percentages in the MCF-7 cell line for MET NP were as follows: 90.66% (IC10), 80.65% (IC20), and 52.25% (IC50). For CXB, the corresponding percentages were 92.06% (IC10), 84.61% (IC20), and 51.63% (IC50). Strikingly, the combination treatment displayed reduced viability percentages: 76.02% (at IC10 of MET and CXB) (*p* < 0.01), 60.86% (at IC20 of MET and CXB) (*p* < 0.01), and 25.22% (at IC50 of MET and CXB) (*p* < 0.01) (Figure 8a). In the case of the MDA-MB-231 cell line, the combined treatment of MET NP and CXB NP yielded viability percentages of 77.35% (at IC10 of MET and CXB) (*p* < 0.05), 62.52% (at IC20 of MET and CXB) (*p* < 0.05), and 31.21% (at IC50 of MET and CXB) (*p* < 0.01) (Figure 8b). The findings of this study indicate that the combined treatment approach resulted in enhanced cytotoxicity, requiring lower systemic drug concentrations to elicit desired responses in breast cancer cells compared to the solo treatments involving MET NP and CXB NP.

### 3.8. Effect of the Combination of MET NP and CXB NP on Cell Viability in 3D Spheroids

**Figure 9:**
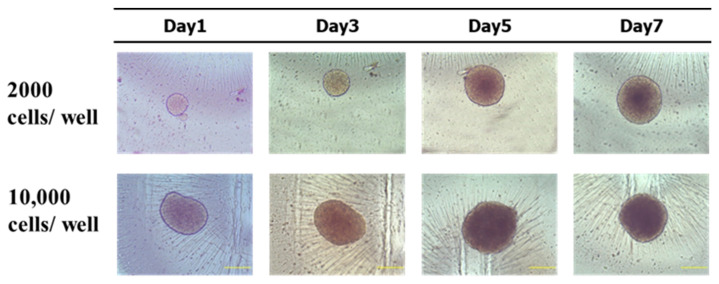
MCF-7 spheroids were cultured over a period of 7 days. The scale bar = 200 µm at 10× objective lens.

Despite promising results in 2D preclinical studies, several drugs have encountered significant failures when translated into clinical settings [32]. Therefore, in order to obtain a clearer picture of the behavior of MET NP and CXB NP in vivo, we used the 3DA spheroid model. The MCF-7 cell line was chosen based on its capacity to generate clearly defined spheroids characterized by a uniform spherical shape and a dense structure (Figure 9). According to our experimental findings, the inclusion of a 20% MC solution as a crowding agent resulted in enhanced cellular aggregation. The cells were seeded at two concentrations, 2000 and 10,000 per well. Both led to the production of spheroids; however, only the concentration of 2000 cells per well exhibited growth in the logarithmic phase throughout a 7-day period. In contrast, the spheroids consisting of 10,000 cells exhibited a phase of growth stabilization by day 5, followed by a subsequent reduction in size. Hence, we opted for the utilization of 2000 cells per well for the viability experiment. On day 7, the diameter of 2000 cells/well spheroids was around 350 ± 50 μm (Figure 9).

**Figure 10:**
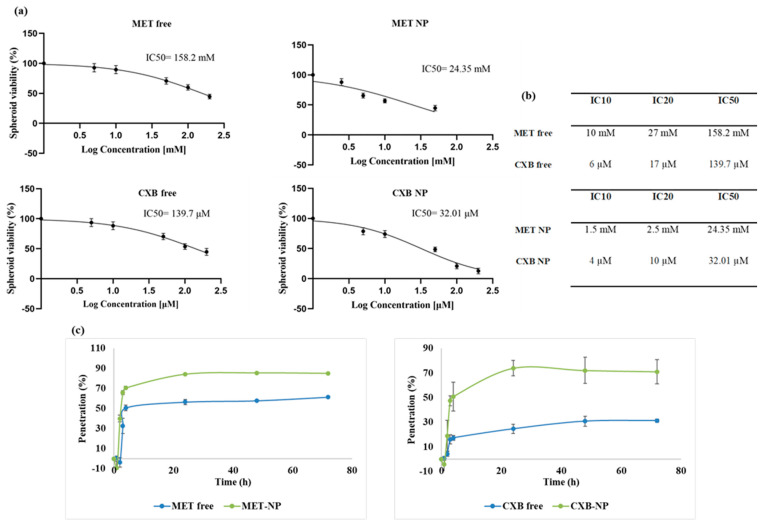
(**a**) The dose-response curve of MET free, MET NP, CXB free, and CXB NP in MCF-7 spheroids and (**b**) a summary of IC10, IC20, and IC50 values. (**c**) Drug penetration curves over a 72 h period for MET free, MET NP, CXB free, and CXB NP in MCF-7 spheroids. Values are the mean of three independent experiments, and error bars represent the standard deviation (SD).

Next, we exposed the 3-day-old spheroids to various concentrations of MET (2.5–200 mM), CXB (5–200 µM), MET NP (2.5–200 mM), and CXB NP (5–200 µM) for 72 h. On day 6, using the Cell Titer-Glo^®^ 3D cell assay, viability was calculated relative to untreated control spheroids, and dose-response curves were constructed to determine the IC10, IC20, and IC50 values (Figure 10a,b).

As expected, we observed that spheroids displayed lower sensitivity to both free drugs and niosomal formulations compared to 2D monolayer cultures. The IC_50_ values for MET as a free drug were significantly higher in MCF-7 3D spheroids (158.2 mM) compared to 2D monolayer cultures (16.1 mM), representing almost nine times the concentration required to achieve the same effect in 2D (Figure 10b). Similar trends were seen for CXB as a free drug (139.7 µM vs. 22.3 µM) and for the niosomal formulations of MET NP (24.35 mM vs. 5.75 mM) and CXB NP (32.01 µM vs. 10.8 µM) in 3D spheroids and 2D monolayers, respectively. The substantial difference in IC50 values can be attributed to the limited drug penetration within the tightly packed spheroids, restricting access to inner senescent and necrotic regions, thereby resulting in reduced cytotoxicity.

However, it was noteworthy that spheroids demonstrated greater sensitivity to niosomes loaded with drugs compared to free drug treatments. For instance, treatment with 50 mM free MET reduced spheroid viability to an average of 70.7%, while the same concentration (50 mM) loaded into niosomes reduced viability to 34.6%. A similar pattern was observed for CXB, with 50 µM free CXB reducing viability to 70.4% on average, whereas niosomes loaded with the same concentration (50 µM) reduced viability to 45.2%. These findings highlight the enhanced efficacy of drug-loaded niosomes in the 3D model.

**Figure 11:**
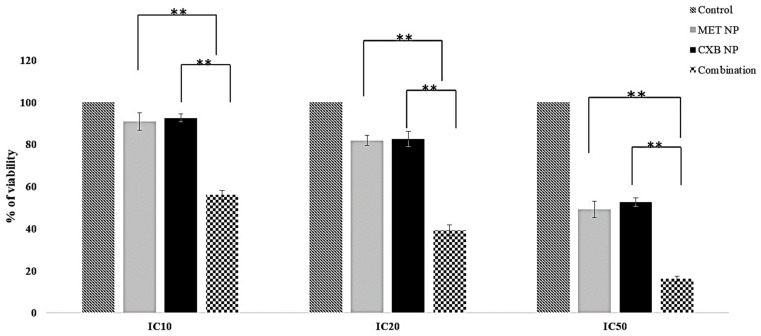
Effect of niosomal combinations of MET and CXB at IC10, IC20, and IC50 on MCF-7 spheroid viability. Values are the mean of three independent experiments, and error bars represent the standard deviation (SD). ** *p* < 0.01.

Given the enhanced response observed in 2D cells when combining MET NP and CXB NP, we evaluated whether this combination would also elicit an improved response in the 3D model (Figure 11). Similar to the combination results in 2D cells, the combination treatment of MET NP and CXB NP resulted in increased inhibition of viable cells compared to each drug alone in the 3D spheroids (*p* < 0.01). The combination of MET NP (IC50 of 24.35 mM) and CXB NP (IC50 of 32.01 µM) exhibited the greatest inhibition of survival, reaching 84%. This combination demonstrated superior efficacy compared to the individual drugs employed alone.

### 3.9. Penetration of MET, MET NP, CXB, and CXB NP

To assess the degree of drug penetration, we employed HPLC to measure the residual drug concentration in the media. This allowed us to indirectly determine the quantity of the drug that had entered the spheroids and accumulated within them. Specifically, we evaluated the penetration and accumulation of MET, MET-NP, CXB, and CXB NP within the spheroids (Figure 10c).

At the initiation of the experiment (Time 0), it was observed that all drugs displayed negligible penetration, providing evidence for the integrity of the spheroid models. In the subsequent hours, both MET NP and CXB NP consistently and significantly exhibited penetration. Specifically, MET NP achieved a penetration rate of 65.93% at the 3 h mark, surpassing the penetration rate of MET, which reached 34.47%. In a comparable manner, CXB NP demonstrated a penetration rate of 47.48% after 3 h, surpassing the 15.92% penetration rate attained by CXB. The aforementioned results emphasize the complicated dynamics of drug penetration and the impact of nanoparticle formulations, providing insights into their prospective utilization in spheroid-focused investigations and drug administration approaches. Furthermore, the sustained and prolonged penetration was observed over 72 h, with MET NP reaching an 85.26% penetration rate compared to METs at 61.50%, and CXB NP demonstrated a 71.08% penetration rate versus 31.29% for CXB, highlighting the therapeutic promise of niosomes for enhanced tissue penetration, which is a crucial aspect to consider in the fields of cancer drug development.

### 3.10. Effect of the Combination Treatment of MET NP and CXB NP on Cell Migration

We conducted an investigation to assess the impact of various formulations on the migration of MCF-7 cells. To ensure the specificity of the treatment’s influence on migration rather than cell death, we chose IC10 for the migration assay. Furthermore, H_2_O_2_ is well-known to induce cell migration at concentrations below (200 µM). Accordingly, we used 200 µM of H_2_O_2_ in the current experiment [18].

**Figure 12:**
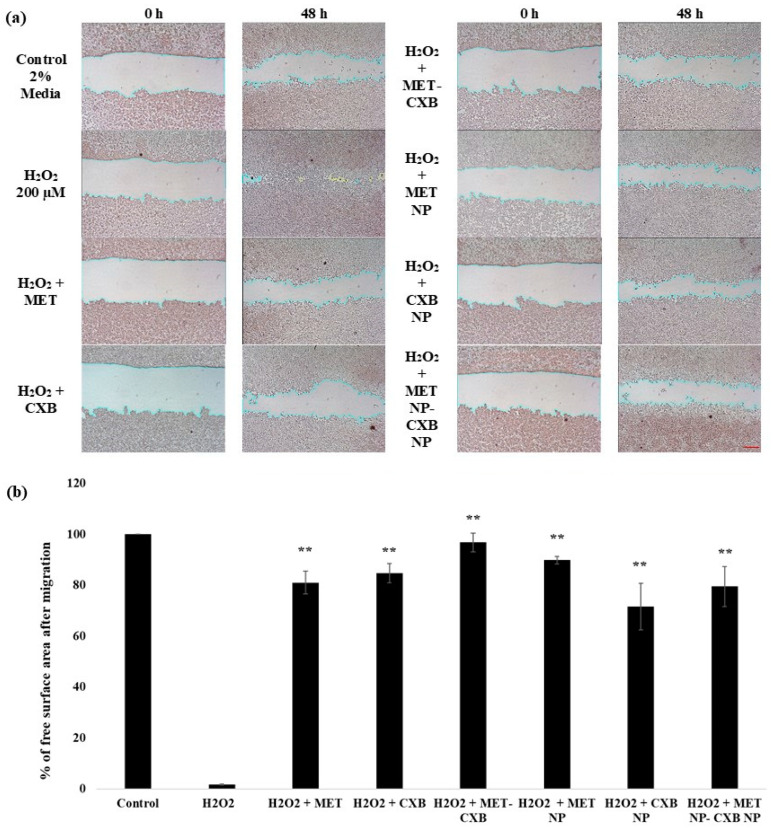
Effect of MET, CXB and MET NP, CXB NP alone and in combinations, on cell migration. (**a**) After cell migration, the free surface area was quantified in two fields per well and then averaged and normalized to the initial free surface area at 0 h. (**b**) The data are presented as the percentage of free surface area after migration. Error bars indicate the standard deviation from three separate experiments, and significance is denoted as ** *p* < 0.01. The scale bar corresponds to 300 μm when using a 10× objective lens.

Our findings revealed that exposure to H_2_O_2_ for 48 h led to a significant increase in MCF-7 cell migration. However, when treated with individual concentrations of MET IC10 (0.3 mM) and CXB IC10 (1 µM), as well as their combination, cell migration induced by H_2_O_2_ was reduced. The reduction in migration was substantial, and the free surface area after treatment was 78%, 81%, and 97%, respectively, compared to 1.7% of the control cells containing H_2_O_2_ (Figure 12). Remarkably, the combination of MET IC10 and CXB IC10 completely halted cell migration in the MCF-7 cell line.

Additionally, we explored the effects of niosomal formulations of MET NP (0.2 mM) and CXB NP (0.7 µM), both individually and in combination, on cell migration. The results demonstrated that MET NP reduced migration by 84% compared to cells treated with H_2_O_2_ (200 µM), while CXB NP reduced migration by 67%. Combining both niosomal formulations resulted in an 80% decrease in cell migration. These findings indicate that both MET and CXB, along with their niosomal formulations, effectively inhibit H_2_O_2_-induced cell migration in MCF-7 cells.

However, it is worth noting that the combined niosomal formulations did not show superior results compared to the combination of free drugs. This difference can be attributed primarily to the slower release of CXB from the niosomes. The release data indicated that approximately 73% of encapsulated CXB was released within 72 h, while only 48.7% of CXB was released during the 48 h timeframe of the migration assay.

## 4. Discussion

This study represents the initial application of MET and CXB in combination for breast cancer treatment. Additionally, it is the first instance of assessing their niosomal formulation in the context of cancer research. Recently, niosomes have attracted significant interest as a promising option for drug delivery systems [33]. These nanoparticles possess distinct characteristics that allow them to encapsulate drugs that are both hydrophilic and lipophilic [13]. This capability has the potential to broaden the range of therapeutic applications for these drugs. Moreover, niosomes demonstrate remarkable attributes such as improving drug distribution, facilitating drug absorption, and providing opportunities for accurate drug targeting [34]. Niosomes are preferred over liposomes as drug delivery methods due to their intrinsic cost-effectiveness, stability, and prolonged shelf life [35] Therefore, niosomes were chosen as the preferred nanocarrier for our dual drug delivery method, instead of traditional liposomes.

The properties of niosomes are significantly influenced by their morphology (i.e., shape and size), and this is crucial for determining their applications (Clauser et al., 2020) [36]. Hence, a thorough characterization was essential to confirm and validate these properties.

The thin-film hydration approach was selected as it was shown in prior studies to consistently generate spherical niosomal particles [11]. The approach involves the utilization of a 1:1 molar ratio of Span 60 and cholesterol. The morphological characteristics of the niosomes were analyzed by the utilization of TEM, revealing a predominantly spherical shape. The circular morphology of these vesicles can be attributed to their inherent stability, mostly resulting from the self-arrangement of surfactants inside the niosomal bilayer [16].

The TEM image of the M2 niosomal formulation showed an average particle size of 111.03 nm, which closely matched the average particle size of 110.6 ± 0.6 nm determined by the particle size analyzer. This is likely due to the low PDI value of 0.139 ± 0.017. The TEM image of the O2 niosomal formulation showed a particle size of 72.06 nm. This size was slightly smaller than the measurement obtained from the particle size analyzer (96.7 ± 0.7 nm) but was still consistent with previous studies [37,38]. The results of our study provide strong evidence that the morphology of the niosomes being studied is consistent and reproducible. This supports the notion that these niosomes can be used as effective nanocarriers for delivering drugs in different applications. The aforementioned sizes and PDIs hold particular significance due to their alignment with the ideal range for nanoparticles, typically within 50 to 200 nm, characterized by a narrow size distribution with PDI below 0.3 for successful cancer targeting [39]. This feature plays a vital role in drug delivery to tumor sites while evading kidney clearance.

Moreover, the significance of HLB values in determining PS is readily evident [40]. Specifically, niosomes formulated with a combination of Span 60 and Tween 60 (HLB: 6.4) exhibited larger PS, measuring 129.5 ± 3.1 nm for M3 and 159.1 ± 1.7 nm for O3, in contrast to niosomes generated solely with Span 60 (HLB: 4.7), which exhibited smaller PS measured at 110.6 ± 0.6 nm for M2 and 96.7 ± 0.7 nm for O2. It has been demonstrated previously that this difference in PS is primarily attributable to the incorporation of additional hydrophilic groups, which increases the niosome surface energy [26].

It is noteworthy that the MET niosomal formulation exhibited a particle size that was relatively greater than the CXB formulations, despite the fact that both formulations contained the same components. This could be explained by the fact that the interaction between hydrophobic drugs and niosome vesicles typically reduces vesicle size. This phenomenon occurs as a result of hydrophobic drugs helping to compact the niosomal structure or promoting the formation of smaller vesicles during preparation [41].

The measurement of ZP is crucial in assessing the stability of niosomes [42]. In this study, MET and CXB niosomes showed ZP values within the optimal range of −41 mV to −58 mV, indicating niosome stability and preventing aggregation. The negative ZP values can be attributed to the presence of free hydroxyl groups in cholesterol and surfactant molecules [43]. Surface charge is a critical consideration in nanocarriers’ potential for effective cancer treatment [44]. Positively charged nanoparticles exhibit increased cellular uptake by tumors, while negatively charged nanoparticles resist protein adsorption, prolonging their circulation and preventing rapid clearance [45,46].

One notable attribute of niosomes is their capacity to effectively encapsulate drug agents [47]. In our research, we investigated the process of encapsulating MET and CXB into niosomes using the thin film hydration method. We used a molar ratio of (1:1) for cholesterol and a non-ionic surfactant. The results showed that the EE% values were excellent and comparable to the findings reported in previous studies [22,48]. The EE% was found to be 64.57 ± 2.02 for M1 and 68.94 ± 1.28 for M2, and the underlying cause for this resemblance can be attributed to the utilization of a common surfactant, namely Span 60, in both formulations. The drop in %EE seen in M3 can be attributed to the formation of hydrogen bonds between the surfactants Tween 80 and Span 60 [13]. The existence of these bonds can increase the stiffness of the niosomal bilayer structure, which may cause the bilayer to break and hinder the vesicle’s ability to efficiently entrap drugs [49]. The formulation of niosomes containing CXB were prepared according to the optimized procedure proposed by Auda et al. [50], which advocated for the use of a 1:1 molar ratio of Span 60 and cholesterol to achieve the highest %EE for CXB. Interestingly, our results demonstrated that the %EE for O2 was notably higher at 94.44 ± 2.09 compared to the findings reported by Auda et al. This increase in %EE was due to an increase in cholesterol content while maintaining a 1:1 molar ratio between Span 60 and cholesterol, as opposed to an increase in cholesterol content per se.

The correlation between %EE and the observed molecular interactions within our niosomes is crucial. The ATR-FTIR analysis revealed the structural properties of our niosomes and their interaction with the niosomal components. Notably, the absence of characteristic peaks for CXB in its niosomal formulations and the minor shifts observed for MET niosomes, as described earlier, indicate effective encapsulation of the drug within the niosomal core rather than the surface as reported, respectively, by Aguilar-Jiménez et al. and Alkilani et al. [10,51].

In addition, our research results show that 89.2% of MET and 77.80% of CXB were released from their respective niosomes under acidic pH conditions compared to 66.8% of MET and 33.9% of CXB at physiological pH. This suggests that there is a possibility of a significant increase in drug release from niosomes when they reach tumor sites, as the lower pH environment facilitates this process [30]. This could lead to the improved targeted delivery of drugs to the tumor, resulting in a stronger therapeutic impact. In accordance with the study aim, we conducted an evaluation of the impact of MET NP and CXB NP on the viability of breast cancer cells using both 2D and 3D cell culture models. The combination of MET and CXB with other chemotherapeutic agents has been widely investigated in many cancer types [5,52,53,54,55]. It has consistently demonstrated that the combination of MET or CXB with chemotherapeutic agents has enhanced efficacy in comparison to therapies involving single-agent therapy. Moreover, it is crucial to acknowledge that the combined use of MET and CXB has been examined in a limited number of studies, predominantly focusing on liver and lung cancer [56,57]. The studies have provided strong evidence of the potential therapeutic advantages of this combined treatment in suppressing cell proliferation and migration.

The findings of our study demonstrated that the application of MET and CXB resulted in a decrease in cell viability in MCF-7 and MDA-MB-231 breast cancer cells. The nanoparticle formulations exhibited superior performance compared to free drugs. In addition, the IC10, IC20, and IC50 values obtained from the analysis indicated that MET NP and CXB NP demonstrated enhanced cytotoxicity at lower concentrations in comparison to their unencapsulated counterparts. This study holds significant importance as it indicates that the utilization of these niosomal formulations could decrease the necessary drug concentrations and, subsequently, mitigate the accompanying adverse effects. The co-administration of MET NP and CXB NP in the 2D cell culture model has led to a notable reduction in cell viability percentages compared to the separate administration of either drug. This suggests the possibility of employing these niosomal formulations in the context of combination therapy for the treatment of breast cancer.

In order to more accurately replicate the tumor environment in vivo, we conducted an assessment of the NPs using a 3D spheroid model. Our observations revealed a reduction in cytotoxicity on the spheroids to both free drugs and niosomal formulations compared to 2D monolayer cultures. This observed expected decrease in sensitivity can be attributed to the limited penetration of drugs into the tightly packed spheroids, hindering their access to the interior regions. Nevertheless, the niosomal formulations demonstrated increased sensitivity in the 3D model in comparison to the free drugs due to enhanced penetration. MET NP exhibited an 85.26% penetration rate, surpassing MET’s 61.50%, and CXB NP demonstrated a 71.08% penetration rate compared to 31.29% for CXB. These results suggest that employing niosomal formulations holds promise in overcoming penetration challenges in 3D culture models, thereby enhancing their effectiveness in mimicking physiological settings.

In addition to evaluating cellular survival, our study aimed to examine the effects of MET, CXB, MET NP, and CXB NP on cell migration, a critical determinant of cancer advancement. The treatments exhibited a reduction in cell migration mediated by H_2_O_2_ in MCF-7 cells. The co-administration of MET and CXB, whether in their free or niosomal formulations, demonstrated significant inhibition of cellular migration. This observation implies that these therapeutic interventions not only affect the survival of cells but also impede the migratory potential of cancer cells, which is a critical factor in the prevention of metastasis.

## 5. Conclusions

In conclusion, the results of our study emphasize the potential of utilizing niosomal formulations of MET and CXB to augment their effectiveness in the treatment of breast cancer for the first time. These formulations have been shown to enhance drug penetration, increase cytotoxicity, and inhibit cell migration, particularly in 3D culture settings. The utilization of MET NP in conjunction with CXB NP exhibits potential as a therapeutic approach in breast cancer. The findings presented in this study provide significant contributions toward the advancement of more efficient and focused treatment strategies for breast cancer patients, harnessing the potential of clinically established drugs.

In addition, our research employing the 3D spheroid model has uncovered the inherent constraints of traditional 2D culture models in accurately forecasting the effectiveness of drugs. This study presented evidence of enhanced response and cytotoxicity when MET NP and CXB NP were combined, as opposed to using the medications individually. This finding underscores the importance of including 3D culture models in drug assessment and combination approaches, as they provide a more precise evaluation of therapeutic efficacy. Our findings pave the way for further investigations in an in vivo setting. Future work should focus on transitioning these promising niosomal formulations into preclinical and clinical trials to assess their safety and efficacy in living organisms. Ultimately, this research could lead to the development of more effective and targeted treatment options for breast cancer patients to improve their clinical outcomes and a better quality of life.

## Figures and Tables

**Table 1:** The composition of MET NP and CXB NP.

Formulation Code	MET (mg)	CXB (mg)	HLB *	Cholesterol (mM)	Span 60 (mM)	Tween 80 (mM)	Hydration volume (mL)
M1	100	-	4.7	75	75	-	15
M2	100	-	4.7	100	100	-	15
M3	100	-	6.4	100	66.6	33.3	15
O1	-	10	4.7	75	75	-	15
O2	-	10	4.7	100	100	-	15
O3	-	10	6.4	100	66.6	33.3	15

* HLB = hydrophilic–lipophilic balance.

**Table 2:** The PS, PDI, ZP, and EE% of MET NP and CXB NP formulation.

Formulation Code	PS (nm) ± SD	PDI ± SD	ZP (mV) ± SD
M1	120.0 ± 1.400	0.180 ± 0.003	−42.15 ± 3.000
M2	110.6 ± 0.600	0.139 ± 0.017	−44.42 ± 1.990
M3	129.5 ± 3.100	0.163 ± 0.007	−56.18 ± 1.890
O1	103.0 ± 1.300	0.316 ± 0.014	−53.93 ± 1.550
O2	96.7 ± 0.700	0.278 ± 0.003	−53.89 ± 5.680
O3	159.1 ± 1.700	0.120 ± 0.020	−50.43 ± 0.785

Results are represented by mean ± SD (n  =  3).

**Table 3:** EE ± SD of the different formulations of MET and CXB.

Formulation Code	EE ± SD (%)
M1	64.57 ± 2.02
M2	68.94 ± 1.28
M3	56.69 ± 3.22
O1	72.19 ± 4.97
O2	94.44 ± 2.09
O3	88.05 ± 3.13

**Table 4:** Characteristic peaks for MET, CXB, Span 60, and cholesterol used in the ATR-FTIR analysis and their corresponding groups.

Characteristic Peak (cm^−1^) of MET	Group Assign to Peak	Characteristic Peak (cm^−1^) of CXB	Group Assign to Peak	Characteristic Peak (cm^−1^) of Span 60	Group Assign to Peak	Characteristic Peak (cm^−1^) of Cholesterol	Group Assign to Peak
3291.5	N–H primary stretching	3336	–NH2 stretching	3400	–OH stretching	3430.5	O-H stretching
3368	N–H primary stretching	3230.5	–NH2 stretching	2917	–CH stretching	2931	C-H stretching
3146.5	N–H secondary stretching	1346	S=O stretching	1736	strong C=O ester bond	2867	C-O bending vibrations
1622.5	C–N stretching	1274.5	-CF3	1174	C–O and C-C stretching vibration	1055	C-O bending vibrations
935.5	N–H out of plane bending	1229	-CF3	721	C–C connections	958.5	aromatic substitutions
736	N–H wagging	1156	S=O stretching			840.5	aromatic substitutions

**Table 5:** Short-term stability study results for M2 and O2 at 4 °C. PS, PDI, and EE were studied as stability parameters.

Formula	Time Interval	PS PS (nm) ± SD	PDI	(%) EE
M2	Freshly prepared	110.6 ± 0.62	0.139 ± 0.01	68.94 ± 1.28
	One month	123.9 ± 1.25	0.157 ± 0.01	66.85 ± 2.06
	Two months	141.4 ± 0.53	0.156 ± 0.02	61.42 ± 1.36
	Three months	149.2 ± 0.70	0.204 ± 0.01	57.12 ± 0.95
O2	Freshly prepared	96.7 ± 0.71	0.278 ± 0.01	94.54 ± 2.09
	One month	102.3 ± 0.47	0.209 ± 0.01	93.81 ± 1.14
	Two months	113.7 ± 0.14	0.189 ± 0.02	91.44 ± 1.67
	Three months	109.7 ± 0.91	0.254 ± 0.08	90.73 ± 3.26

Results are represented by mean  ±  SD (n  =  3).

**Table 6:** IC Values of MET and CXB with their nanoparticle formulations in MCF-7 and MDA-MB-231 breast cancer cell lines.

	**MET**			**CXB**		
	IC10	IC20	IC50	IC10	IC20	IC50
MCF-7	0.39 mM	1.28 mM	17.14 mM	1.39 µM	5.00 µM	22.30 µM
MDA-MB-231	5.00 mM	8.00 mM	24.20 mM	12.59 µM	18.17 µM	28.10 µM
	**MET NP**			**CXB NP**		
	IC10	IC20	IC50	IC10	IC20	IC50
MCF-7	0.21 mM	0.60 mM	5.75 mM	0.66 µM	1.42 µM	10.82 µM
MDA-MB-231	2.89 mM	5.00 mM	16.10 mM	5.05 µM	9.00 µM	16.34 µM

## Data Availability

The data presented in this study are available upon request from the corresponding author.

## Supplementary Materials

The following supporting information can be downloaded at https://www.mdpi.com/article/10.3390/cancers15205004/s1, Figure S1. In vitro drug release of (a) MET NP (M2) and (b) CXB NP (O2) at pH (7.4).
