# Supplementary figures and images for: Niosomal Delivery of Celecoxib and Metformin for Targeted Breast Cancer Treatment
Source: Cancers (Basel). 2023 Oct 16;15(20):5004. doi: 10.3390/cancers15205004 (PMC10605450; doi:10.3390/cancers15205004)

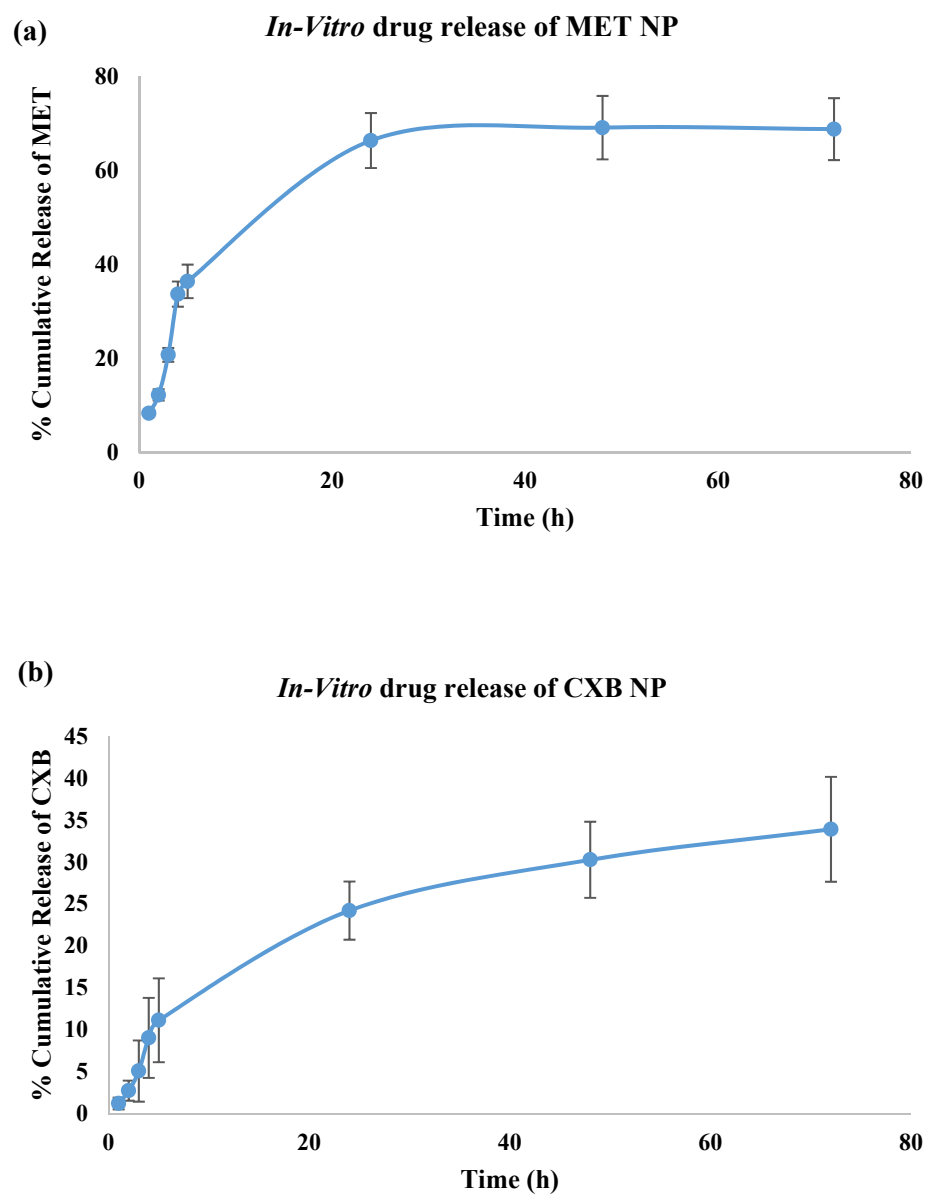

**Figure S1.** *In vitro* drug release of (a): MET NP (M2) and (b): CXB NP (O2) at pH (7.4).

Supplement: Supplementary file 1 [file cancers-15-05004-s001.zip › cancers-2637518-supplementary.pdf]
